# Role of the deubiquitinating enzyme UCH-L1 in mitochondrial function

**DOI:** 10.3389/fncel.2023.1149954

**Published:** 2023-03-23

**Authors:** Alexandre Bouron, Laurence Aubry, Desirée Loreth, Marie-Odile Fauvarque, Catherine Meyer-Schwesinger

**Affiliations:** ^1^Université Grenoble Alpes, Inserm, CEA, UA13, BGE, Grenoble, France; ^2^Institute of Cellular and Integrative Physiology, University Medical Center Hamburg-Eppendorf, Hamburg, Germany

**Keywords:** mitochondria, brain, deubiquitinating enzymes, neurodegeneration, pathology, UCH-L1

## 1. Introduction: Identification of UCH-L1 (PGP9.5)

High-resolution two-dimensional electrophoresis was employed to detect soluble protein gene products (PGPs) in aqueous extracts from human brain and twelve other organs (Jackson and Thompson, 1981). This permitted the identification of four specific brain PGPs, one of them termed PGP9.5. Its molecular identity was unknown at the time but PGP9.5 was described as a “*soluble nervous system-specific protein*” (Jackson and Thompson, 1981) albeit found at much lower levels in other organs like kidney, large intestine, prostate and testis. The PGP9.5 protein was shown to display COOH-terminal hydrolase activity (Wilkinson et al., 1989) and finally turned out to correspond to ubiquitin carboxyl-terminal hydrolase L1 (UCH-L1), an enzyme previously isolated from cytosolic fractions of bovine calf thymus (Duerksen-Hughes et al., 1989; Mayer and Wilkinson, 1989). Immunoperoxidase staining of human cerebral cortex sections showed the restricted presence of UCH-L1 (PGP9.5) in neurons (Doran et al., 1983). Its brain concentration was at least 50 times greater than in other organs like kidney, prostate, or testis. Doran et al. (1983) introduced UCH-L1 as a “*new neurone-specific cytoplasmic marker*” thought to represent 1–2% of total brain soluble proteins. Additional studies extended these observations by showing that UCH-L1 was not only present in neurons of the central nervous system but also in neurons of the peripheral nervous system and in cells of the neuroendocrine system (Thompson et al., 1983). UCH-L1 (PGP9.5) (mRNA and protein) was detected at very early stages (e.g., 10 days p.c.) of neural tube formation (Schofield et al., 1995) in progenitor cells and neurons (Kent and Clarke, 1991).

## 2. UCH-L1 subcellular localization

Although initially described as a major protein of the cytosol, nearly 30% of neuronal UCH-L1 is membrane-associated (Bishop et al., 2014). It is now clear that a significant portion of UCH-L1 is associated to membranes of the endoplasmic reticulum (ER) (Liu et al., 2009; Gao et al., 2020). Immunogold labeling experiments on rat proximal caput epididymidis had previously revealed a mitochondrial localization (Martin et al., 1995). The association of UCH-L1 with mitochondria has however been questioned. For instance, the presence of UCH-L1 in mitochondrial-enriched fractions isolated from brain was proposed to result from a contamination with other fractions such as synaptosomes (Van Laar et al., 2009). Subsequent studies provided novel arguments in favor of a link between UCH-L1 and mitochondria. (i) A truncated variant of UCH-L1 (NT-UCH-L1) identified in the brain was found to associate with mitochondria (Kim et al., 2014). (ii) Low levels of the enzyme were detected in mitochondrial fractions prepared from soleus, a skeletal muscle that preferentially contains slow oxidative muscle fibers (Gao et al., 2020). (iii) Immunofluorescence stainings showed a close association of UCH-L1 with ER and mitochondria in muscle and neuroblastoma SH-SY5Y cells (Cerqueira et al., 2020; Gao et al., 2020). (iv) The DUB was shown to influence the morphology and respiratory functions of mitochondria in skeletal muscle (Gao et al., 2020). Altogether, these experimental data point toward the existence of a link between UCH-L1 and the vital organelles mitochondria.

## 3. Molecular and cellular functions of UCH-L1

UCH-L1 is a 223 amino acids protein (25 kDa) belonging to the Ub carboxyl-terminal hydrolases (UCHs) subfamily of DUBs that comprises three other members: UCH-L3, UCH-L5 (UCH37) and BAP1 (BRCA1-associated protein 1). So far, three main functions have been associated to this protein (Mi and Graham, 2023). UCH-L1 can generate free Ub species, thereby contributing to the recycling of Ub. Intriguingly, it also exhibits a Ub E3 ligase activity (Liu et al., 2002). However, the main cellular function of UCH-L1 is thought to be related to its binding to monomeric Ub that prevents the proteasomal degradation of the polypeptide. Thus, by maintaining a pool of available Ub within cells, UCH-L1 is a key regulator of the multiple cellular Ub-dependent biological processes. In addition, altered protein expression levels of UCH-L1 have been observed in a wide range of tumors (Jara et al., 2013; Sharma et al., 2020).

Originally introduced as a specific neuronal brain protein, mutations in the human UCH-L1 protein, animal models and pharmacological studies indicate that the loss of UCH-L1 or the impairment of its enzymatic activity is associated with neurodegenerative phenotypes (Mi and Graham, 2023). UCH-L1 seems required to maintain the integrity of synaptic structures as shown in the hippocampus (Cartier et al., 2009) and at the neuromuscular junction (Chen et al., 2010). UCH-L1 has also been introduced as a promising serum biomarker of neuronal cell injury in patients with traumatic brain injury (Papa et al., 2016). However, a detailed molecular understanding of the neuronal functions of UCH-L1 in health and disease is still lacking.

## 4. UCH-L1 and mitochondrial proteins and functions

As mentioned above, a fraction of UCH-L1 has been shown to localize to mitochondria. In parallel, several studies have documented a functional link between the DUB and these organelles. Studies conducted on neuroblastoma SH-SY5Y and immortalized INS1 beta cells showed that silencing of UCH-L1 expression (*via* a shRNA strategy) induces significant changes in mitochondrial morphology and reduces interactions (tethering) between mitochondria and the ER by increasing the physical distance between the two organelles (Cerqueira et al., 2020). In addition, UCH-L1 was shown to associate with mitochondrial proteins. Immunoprecipitation experiments identified the mitochondrial chaperone protein HSP60 as a partner of UCH-L1 in skeletal muscle from mice (Gao et al., 2020). Tandem affinity purification coupled to mass spectrometry showed that UCH-L1 can also form a complex with Parkin, an E3 Ub ligase involved in mitophagy, when overexpressed in HEK-293 cells (Davison et al., 2009), suggesting that UCH-L1 may participate to the clearance of dysfunctional mitochondria.

Another interesting feature of UCH-L1 is its impact on the abundance of various mitochondrial proteins. For instance, diminishing and increasing UCH-L1 expression have been shown to reduce and enhance mitofusin protein levels, respectively (Cerqueira et al., 2020). However, UCH-L1 has been proposed to regulate mitochondrial physiology beyond mitophagy (Cerqueira et al., 2020). Deletion of UCH-L1 in the brain of mice increases the protein levels of the manganese (Mn) superoxide dismutase (MnSOD) and the ATP synthase subunit beta (ATPB), two mitochondrial matrix proteins that exert antioxidant and ATP production functions, respectively (Reinicke et al., 2019). In skeletal muscles from mice, the loss of UCH-L1 decreases the abundance of SBHD (component of complex II) and UQCRC2 (component of complex III) proteins, whereas it increases the protein abundance of NDUFB8 (a protein of complex I) (Gao et al., 2020), all three proteins being components of the mitochondrial respiratory chain. Of note, deletion of the *Uch-l1* gene (*dUch*) in *Drosophila* brain elevates the mRNA levels of the cytosolic and mitochondrial SOD (Huynh et al., 2022), an observation supporting a conserved functional relationship between UCH-L1 and mitochondrial proteins in fly and mammals.

Direct measurements of mitochondrial activity have demonstrated that UCH-L1 interferes with mitochondrial bioenergetics. In SH-SY5Y and immortalized INS1 beta cells, UCH-L1 silencing augments the activity of the mitochondrial complex I, the mitochondrial ATP production and oxygen consumption (Cerqueira et al., 2020). In mouse muscle cells, the lack of UCH-L1 reduces the activity of the mitochondrial electron transfer chain complex II/III (Gao et al., 2020), which is likely to perturb the mitochondrial production of ATP. In *Drosophila* brains, deletion of *dUch* causes a severe reduction (>50%) of the ATP levels without modifying the mitochondrial membrane potential (Huynh et al., 2022). In contrast, the pharmacological inhibition of UCH-L1 activity with LDN-57444 has been shown to reduce the mitochondrial membrane potential of oocytes (Yuan et al., 2020).

A pathophysiological connection between UCH-L1 and mitochondria has been established in Alzheimer's and Parkinson's diseases, two prominent neurodegenerative disorders. A significant fraction of UCH-L1 colocalizes with Parkin and mitochondria *in vivo* in Alzheimer's disease brains as well as in transgenic Alzheimer's disease mice (Corsetti et al., 2015). In association with Parkin, UCH-L1 promotes neuronal death by causing an extensive mitochondrial clearance (Corsetti et al., 2015). Nakamura et al. (2021) demonstrated another role of UCH-L1 in Alzheimer's disease: the DUB can function as an upstream signaling molecule participating in an intracellular cascade involving Cdk5 and leading to the transfer of nitric oxide (NO) to the mitochondrial GTPase Drp1 to form NO-Drp1. This non-canonical pathway has been proposed to stimulate mitochondrial fragmentation and bioenergetics dysregulation leading to synaptic insults (Nakamura et al., 2021). The UCH-L1-dependent transnitrosylation pathway could play a role in Alzheimer's disease-related synapse loss and in the pathogenesis of neurodegenerative disorders (Nakamura et al., 2021). Additionally, Ham et al. (2021) showed that UCH-L1 could play a role in Parkinson's disease by (indirectly) controlling mitochondrial homeostasis. UCH-L1 loss promotes mitophagy in *Drosophila* mutants and mammalian cell lines, probably *via* a process involving pyruvate kinase, a key glycolytic enzyme. Indeed, depletion of UCH-L1 strongly decreases the amount of pyruvate kinase by favoring its proteasomal degradation. Altogether, UCH-L1 seems to exert a house-keeping role in mitochondrial homeostasis that depends on the cytosolic energy status (Ham et al., 2021). Moreover, the authors showed that loss of UCH-L1 rescues some defects related to Parkinson's disease. Taken together, these data indicate that UCH-L1 could participate in the regulation of glucose metabolism, mitochondrial homeostasis, and Parkinson's disease pathogenesis (Ham et al., 2021). This link between mitochondria and UCH-L1 has been reinforced by Roy et al. (2022) who showed another aspect of this complex regulation: the pesticide rotenone inhibits the electron transport chain complex I of neuroblastoma N2A cells, enhancing the ROS production while decreasing cellular ATP levels and collapsing the mitochondrial membrane potential. Importantly, rotenone strongly diminishes UCH-L1 mRNA and protein levels (Roy et al., 2022). This observation suggests the intriguing possibility of the existence of a bidirectional regulation of mitochondria and UCH-L1: UCH-L1 influences mitochondrial activity while mitochondria regulate the expression of UCH-L1.

## 5. Conclusion

**Figure 1:**
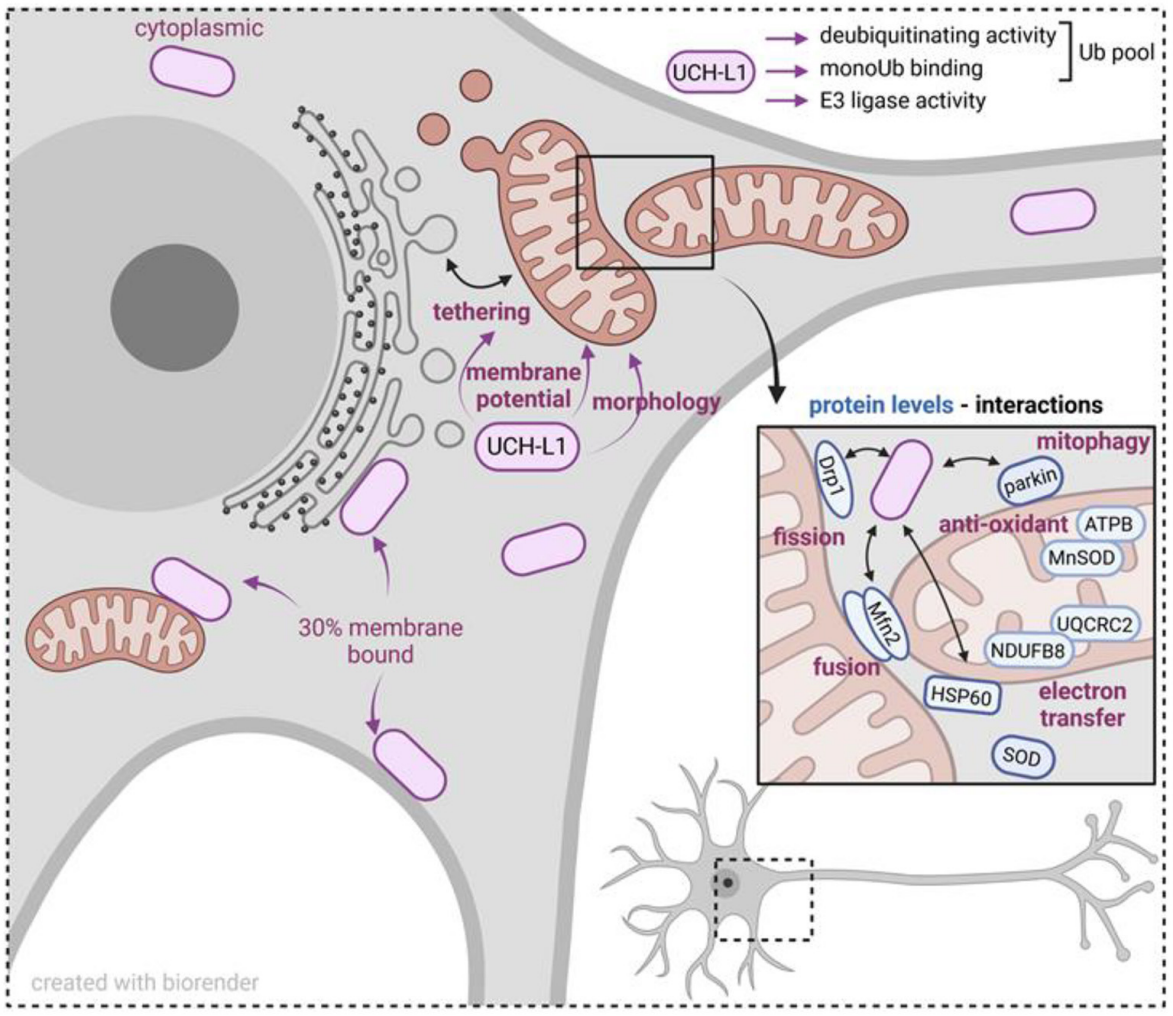
Scheme summarizing mitochondrial proteins and mitochondrial functions influenced by UCH-L1. UCH-L1 is the most abundant deubiquitinating enzyme within neurons, predominantly in the cytoplasm, and 30% as a membrane bound protein. Biochemically, UCH-L1 regulates the cytoplasmic pool of mono ubiquitin (Ub) and exerts an E3 ligase activity. UCH-L1 localizes to the mitochondrial membrane and is associated with modifying mitochondrial-ER tethering, mitochondrial membrane potential and morphology. UCH-L1 impacts the abundance of mitochondrial cytoplasmic and matrix proteins and by this influences mitochondrial fusion and fission processes, electron transfer, and removal of defective mitochondria by mitophagy. In summary, UCH-L1 affects mitochondrial homeostasis and cellular energy status.

Altogether, these studies point toward a complex and multifaceted evolutionary conserved role of UCH-L1 in mitochondrial functions (Figure 1). Understanding the molecular mechanisms at work is a promising and exciting avenue for future studies. They should bring important information on the pathophysiological relevance of this DUB in neuronal physiology and health and disease states.

## Author contributions

AB, LA, DL, M-OF, and CM-S: bibliographic search, manuscript writing, and editing. All authors contributed to the article and approved the submitted version.
